# Hepatic n-3 Polyunsaturated Fatty Acid Depletion Promotes Steatosis and Insulin Resistance in Mice: Genomic Analysis of Cellular Targets

**DOI:** 10.1371/journal.pone.0023365

**Published:** 2011-08-10

**Authors:** Barbara D. Pachikian, Ahmed Essaghir, Jean-Baptiste Demoulin, Audrey M. Neyrinck, Emilie Catry, Fabienne C. De Backer, Nicolas Dejeans, Evelyne M. Dewulf, Florence M. Sohet, Laurence Portois, Louise Deldicque, Olivier Molendi-Coste, Isabelle A. Leclercq, Marc Francaux, Yvon A. Carpentier, Fabienne Foufelle, Giulio G. Muccioli, Patrice D. Cani, Nathalie M. Delzenne

**Affiliations:** 1 Metabolism and Nutrition Research Group, Louvain Drug Research Institute, Université Catholique de Louvain, Brussels, Belgium; 2 De Duve Institute, Université Catholique de Louvain, Brussels, Belgium; 3 Toxicology and Cancer Biology Research Group, Louvain Drug Research Institute, Université Catholique de Louvain, Brussels, Belgium; 4 Laboratory of Experimental Surgery, Université Libre de Bruxelles, Brussels, Belgium; 5 Research Centre for Exercise and Health, Department of Biomedical Kinesiology, Katholieke Universiteit Leuven, Leuven, Belgium; 6 Laboratory of Hepato-Gastroenterology, Institut de Recherche Expérimentale et Clinique, Université Catholique de Louvain, Brussels, Belgium; 7 Research Group in Muscle and Exercise Physiology, Institute of Neuroscience, Université Catholique de Louvain, Louvain-la-Neuve, Belgium; 8 INSERM, UMR-S 872, Centre de Recherche des Cordeliers, Paris, France; 9 Bioanalysis and Pharmacology of Bioactive Lipids lab, CHAM7230, Louvain Drug Research Institute, Université Catholique de Louvain, Brussels, Belgium; Paris Institute of Technology for Life, Food and Environmental Sciences, France

## Abstract

Patients with non-alcoholic fatty liver disease are characterised by a decreased n-3/n-6 polyunsaturated fatty acid (PUFA) ratio in hepatic phospholipids. The metabolic consequences of n-3 PUFA depletion in the liver are poorly understood. We have reproduced a drastic drop in n-3 PUFA among hepatic phospholipids by feeding C57Bl/6J mice for 3 months with an n-3 PUFA depleted diet (DEF) versus a control diet (CT), which only differed in the PUFA content. DEF mice exhibited hepatic insulin resistance (assessed by euglycemic-hyperinsulinemic clamp) and steatosis that was associated with a decrease in fatty acid oxidation and occurred despite a higher capacity for triglyceride secretion. Microarray and qPCR analysis of the liver tissue revealed higher expression of all the enzymes involved in lipogenesis in DEF mice compared to CT mice, as well as increased expression and activation of sterol regulatory element binding protein-1c (SREBP-1c). Our data suggest that the activation of the liver X receptor pathway is involved in the overexpression of SREBP-1c, and this phenomenon cannot be attributed to insulin or to endoplasmic reticulum stress responses. In conclusion, n-3 PUFA depletion in liver phospholipids leads to activation of SREBP-1c and lipogenesis, which contributes to hepatic steatosis.

## Introduction

Dietary n-3 polyunsaturated fatty acid (PUFA) have important metabolic effects due to their involvement in eicosanoid biosynthesis and their ability to modulate the transcription of regulatory genes [1], [2]. Numerous in vitro and in vivo studies have demonstrated that n-3 PUFA are able to coordinate both the upregulation of lipid oxidation and the downregulation of lipid synthesis. On the one hand, n-3 PUFA promote fatty acid oxidation by binding and activating peroxisome proliferator-activated receptor α (PPARα) [3], [4]. On the other hand, n-3 PUFA suppress lipogenesis by inhibiting sterol regulatory element binding protein-1c (SREBP-1c) gene expression and/or activation by proteolysis [4]–[6].

Several clinical studies have reported the beneficial effects of n-3 PUFA (mostly eicosapentaenoic and/or docosahexaenoic acids from marine origin) supplementation on triglyceridemia [7], blood pressure [8], inflammation [9] and insulin sensitivity [10].

Of interest, the western type diet is low in n-3 PUFA and brings excessive amount of n-6 PUFA, leading to an imbalanced n-3/n-6 PUFA intake compared to the dietary recommendations. High dietary n-6 PUFA and low dietary n-3 PUFA intake has been suggested to promote the pathogenesis of several diseases, including cardiovascular disease, inflammatory and autoimmune disease [11], [12]. Moreover, a lower intake in dietary sources of n-3 PUFA was suggested to be associated with non-alcoholic fatty liver disease [13], [14]. In fact, patients with hepatic steatosis present a lower n-3/n-6 PUFA ratio in liver tissue biopsies, namely in phospholipids subfractions, and in red blood cells [15], [16]. In accordance with this observation, rats and mice presenting a depletion of n-3 PUFA for two generations display several features of metabolic syndrome including hepatic steatosis [17], [18]. However, the biochemical mechanisms explaining the hepatic alterations occurring upon n-3 PUFA depletion remain unclear.

Therefore, in the present study, we have investigated, in mice, the effect of n-3 PUFA depletion established for 3-months on hepatic lipid composition and metabolism using molecular and integrative physiological approaches in vitro and in vivo. We observed a stimulation of the hepatic lipogenic pathway, most likely induced by the increased expression and activity of SREBP-1c.

## Results

### Mice fed with a diet depleted in n-3 PUFA exhibit a decrease in n-3 PUFA in hepatic phospholipid fractions and changes in hepatic endocannabinoid content

DEF mice exhibited a large drop in n-3 PUFA content in hepatic phospholipids (PLs), thereby confirming the tissue depletion (Table 1). Despite a qualitative change in n-6 PUFA in favour of arachidonic acid (C20:4 n-6), the total amount of n-6 PUFA in hepatic PLs was similar between both groups. The other modification observed in DEF mice was a 51% increase in the content of monounsaturated fatty acid (MUFA) and especially of oleic acid (C18:1) compared to CT mice (Table 1). In accordance with the changes in arachidonic and oleic acid, we found a higher level of bioactive lipids belonging to the endocannabinoid system and known to control lipogenesis, namely 2-arachidonoylglycerol (2-AG) and *N*-oleoylethanolamine, in the liver of DEF mice than in CT mice. However, for *N*-oleoylethanolamine it was not significant (Table S1).

**Table 1:** Fatty acids pattern in liver phospholipids (PLs) fractions of CT and DEF mice.

µg/g liver tissue	CT	DEF
C14:0	31.2	39.1
C16:0	5842.0	4961.6
C18:0	3208.0	2900.5
C20:0	43.7	40.9
C22:0	153.8	142.0
C24:0	109.9	91.7
C16:1 n-7	331.5	384.2
C18:1 n-9	1758.3	2775.8
C20:1 n-9	53.3	76.8
C18:3 n-3	52.4	0.1
C18:4 n-3	ND	ND
C20:5 n-3	84.9	0.1
C22:3 n-3	ND	ND
C22:5 n-3	148.6	19.2
C22:6 n-3	4612.0	1407.0
C18:2 n-6	4527.6	3314.9
C18:3 n-6	76.3	65.6
C20:2 n-6	57.1	44.6
C20:3 n-6	355.1	423.9
C20:4 n-6	6068.3	6728.7
C22:4 n-6	78.3	87.1

Data are values determined in hepatic PLs of a pool of 4 fasted mice fed with control diet (CT) and of a pool of 4 fasted mice fed an n-3 PUFA depleted diet (DEF) for 3 months.

ND  =  non detectable.

### n-3 PUFA depletion decreases fatty acid oxidation and promotes hepatic lipid synthesis, storage and secretion

**Figure 1:**
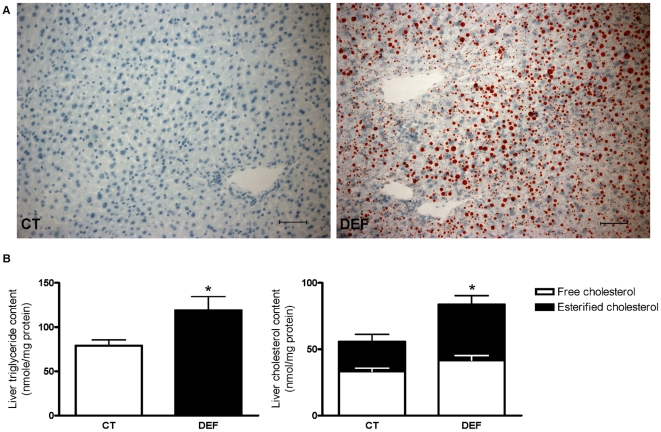
Accumulation of lipids in the livers of n-3 PUFA depleted mice. Oil red staining performed on frozen liver sections of fasted mice fed a control (CT) or n-3 PUFA-depleted (DEF) diet for 3 months. Bar = 50 µm (A). TG, free cholesterol and esterified cholesterol content in the livers of mice fed a control (CT) or n-3 PUFA-depleted (DEF) diet for 3 months. Data are the mean ± SEM. ^*^: mean values significantly different (P<0.05, Student's *t*-test) (B).

Histological analysis revealed a higher number and size of lipid droplets in the livers of DEF mice compared to CT mice (Figure 1A). These observations were confirmed by the biochemical analysis showing that lipid accumulation in the liver of DEF mice was due to an increased hepatic content of triglycerides (TG) and esterified cholesterol (Figure 1B).

**Figure 2:**
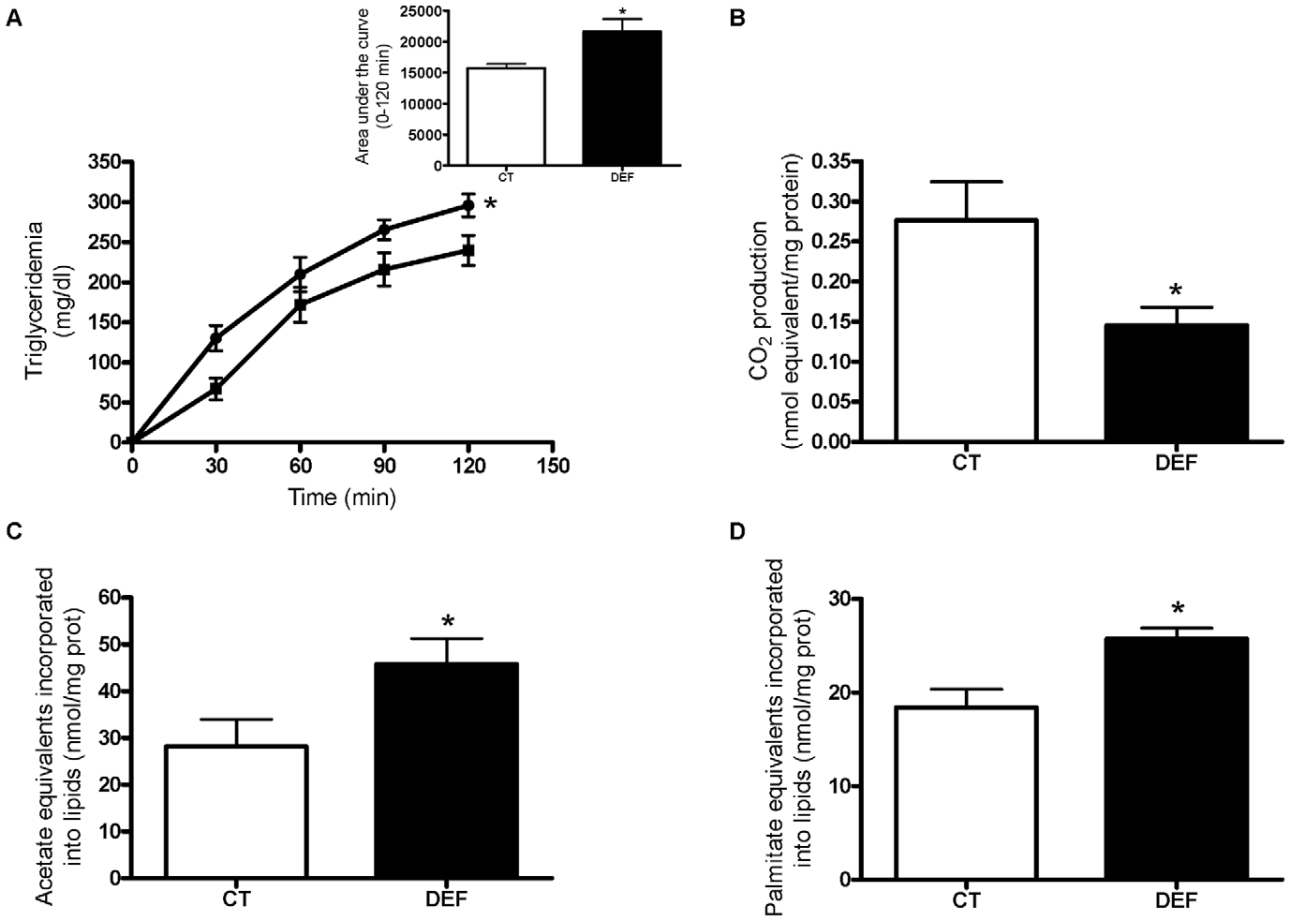
n-3 PUFA depletion leads to decreased hepatic fatty acid oxidation and increased TG secretion and synthesis. Mice were fed a control (CT) or n-3 PUFA-depleted (DEF) diet for 3 months. To measure hepatic TG secretion, fasted CT mice (closed squares, n = 4) and DEF mice (closed circles, n = 6) were injected with tyloxapol (0.5 mg tyloxapol/g body weight). Data are the mean ± SEM. ^*^: mean values significantly different (P<0.05, two-way ANOVA; for the area under the curve, P<0.05, Student's *t*-test) (A). Precision-cut liver slices (PCLSs) obtained from fasted CT (n = 8) and DEF (n = 6) mice were incubated with [^14^C]-palmitate for 3 hours to measure CO_2_ produced from fatty acid oxidation (B). TG synthesis from [^14^C]-acetate (C) and fatty acid esterification into TG from [^14^C]-palmitate (D) were measured in PCLSs obtained from fed CT (n = 7) and DEF mice (n = 8). Data are the mean ± SEM. ^*^: mean values significantly different (P<0.05, Student's *t*-test).

DEF mice exhibited a higher hepatic TG secretion than CT mice, as measured following lipoprotein lipase inhibition (tyloxapol injection) (Figure 2A). Precision-cut liver slice (PCLS) is a model that allows preservation of the liver lobule architecture by maintaining cell diversity and interactions. PCLSs prepared from fasted mice were incubated with [^14^C]-palmitate to measure ^14^CO_2_ release during incubation. By this method, we found that the livers of DEF mice presented lower fatty acid oxidation compared to those of CT mice (Figure 2B). Incubation of PCLSs from fed CT and DEF mice with [^14^C]-acetate or [^14^C]-palmitate showed a 40% increase in TG synthesis and FA-esterification into TG in DEF mice compared to CT mice (Figure 2C, D).

### Microarray analysis confirms a metabolic shift in favour of fatty acid and cholesterol synthesis at the expense of fatty acid oxidation in the livers of n-3 PUFA depleted mice

**Figure 3:**
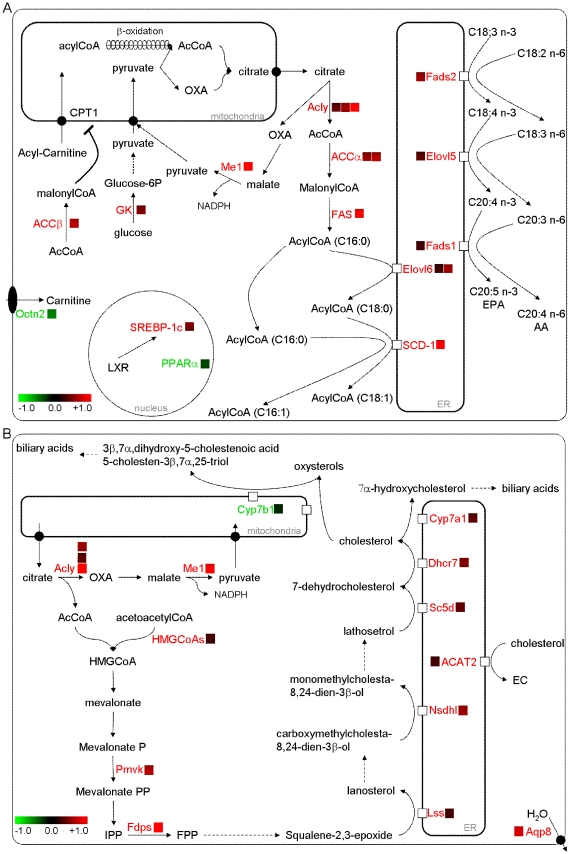
n-3 PUFA-depleted mice exhibited higher mRNA content of enzymes involved in fatty acid and cholesterol synthesis. n-3 PUFA depletion induces increased hepatic expression of enzymes involved in lipogenesis (A) and cholesterol synthesis (B). Microarray analysis was performed on a pool of RNA obtained from fasted mice fed a control (CT) or n-3 PUFA-depleted (DEF) diet for 3 months. In red are depicted enzymes and factors for which mRNA content in the liver of DEF mice was higher and in green those for which mRNA content was lower, than the values measured in CT mice in the fasted state. The color bare refers to the log_2_ fold change in DEF mice versus value obtained in CT mice. No threshold was applied on the fold change. We applied a statistical significance threshold calculated by the MAS5 algorythm as recommended by the manufacturer (Affymetrix). EC  =  esterified cholesterol. See Table S2 for raw data.

The gene expression profiles in the livers of CT and DEF mice were analysed by microarray in both the fasted and fed state. Increased expression of all of the enzymes involved in fatty acid synthesis and desaturation was observed in DEF mice compared to CT mice in the fed and fasted states (Figure 3A and Table S2). The statistical significance of this observation was confirmed by analysing gene ontology with the bioinformatics tool DAVID (Table S3). DEF mice exhibited a lower expression of several markers of fatty acid oxidation, including PPARα and its target genes (Figure 3A and Table S2). The bioinformatics evaluation of transcription factor target genes among the list of genes regulated using TFactS [19] supported the inhibition of the PPARα pathway (Table S4).

In agreement with the physiological data, phospholipid transfer protein (PLTP) and Apolipoprotein B (Apo B), two factors involved in fatty acid secretion, were increased in DEF mice when compared to CT mice (Table S2).

Increased expression of several enzymes involved in cholesterol synthesis (from HMGCoAs to Dhcr7) and esterification for hepatic secretion (ACAT2) also occurred in fed and fasted DEF mice compared to CT mice (Figure 3B and Table S2). With regard to bile acid metabolism, a higher expression of Cyp7a1 and a lower expression of Cyp7b1 suggest a stimulation of the classic bile acid synthesis pathway at the expense of the alternative pathway through n-3 PUFA depletion (Figure 3B and Table S2).

### SREBP-1c is involved in the metabolic alterations occurring in the livers of n-3 PUFA depleted mice

**Figure 4:**
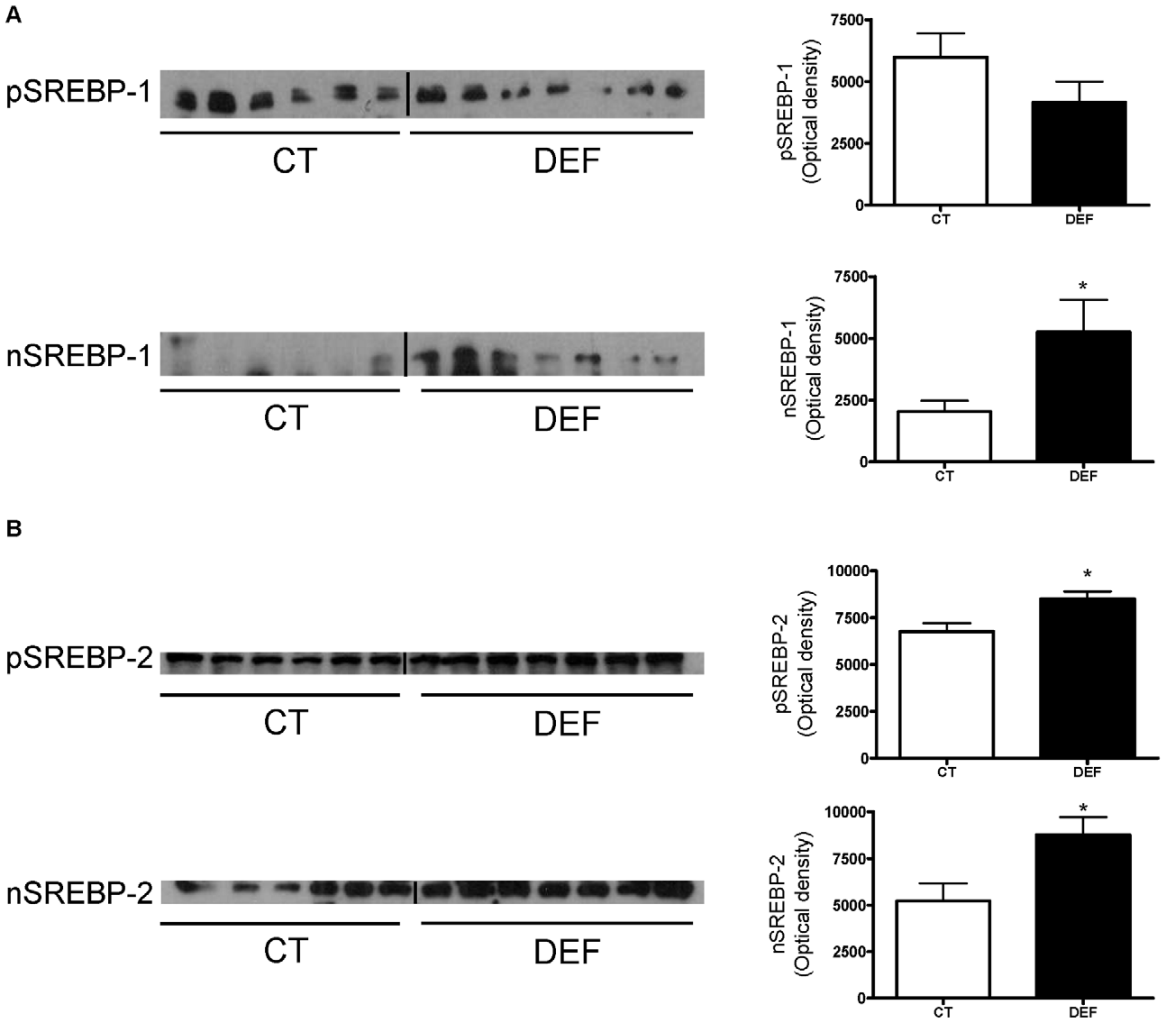
n-3 PUFA depletion leads to hepatic SREBP-1 and SREBP-2 activation. Liver immunoblot analysis of the hepatic SREBP-1 precursor (pSREBP-1) and nuclear (nSREBP-1) form (A) and of the hepatic SREBP-2 precursor (pSREBP-2) and nuclear (nSREBP-2) form (B) from fasted mice fed a control (CT; n = 6) or n-3 PUFA depleted (DEF; n = 7) diet for 3 months. Quantification of immunoblots is shown at right. Data are the mean ± SEM. ^*^: mean values significantly different (P<0.05, Student's *t*-test).

TFactS analysis of microarray results revealed activation of SREBPs (Table S4) but did not distinguish between SREBP-2, SREBP-1c and SREBP-1a. Microarray analysis showed a higher mRNA content of SREBP-1, a key transcription factor involved in the regulation of lipogenesis, in the livers of DEF mice than in those of CT mice (Figure 3A and Table S2). As SREBP-1c and SREBP-2 are the main forms present in the liver, it could be assumed that SREBP-1c was responsible for the higher SREBP-1 expression observed by the microarray. In fact, the higher expression of SREBP-1c and two major enzymes involved in fatty acid synthesis (FAS and SCD-1) was confirmed by qPCR (Table S5). The western blot analysis of SREBP-1 in the cytoplasmic and nuclear fractions revealed a higher hepatic content of nuclear SREBP-1 at the expense of the precursor form in the livers of DEF mice compared to those of CT mice (Figure 4A). It should be noted that the content of the SREBP-2 precursor and mature form were also higher in the livers of DEF mice than in those of CT mice (Figure 4B).

Hence, we further analysed several metabolic pathways involved in the regulation of SREBP-1c expression and activation.

### Mice depleted of n-3 PUFA display hepatic insulin resistance

**Figure 5:**
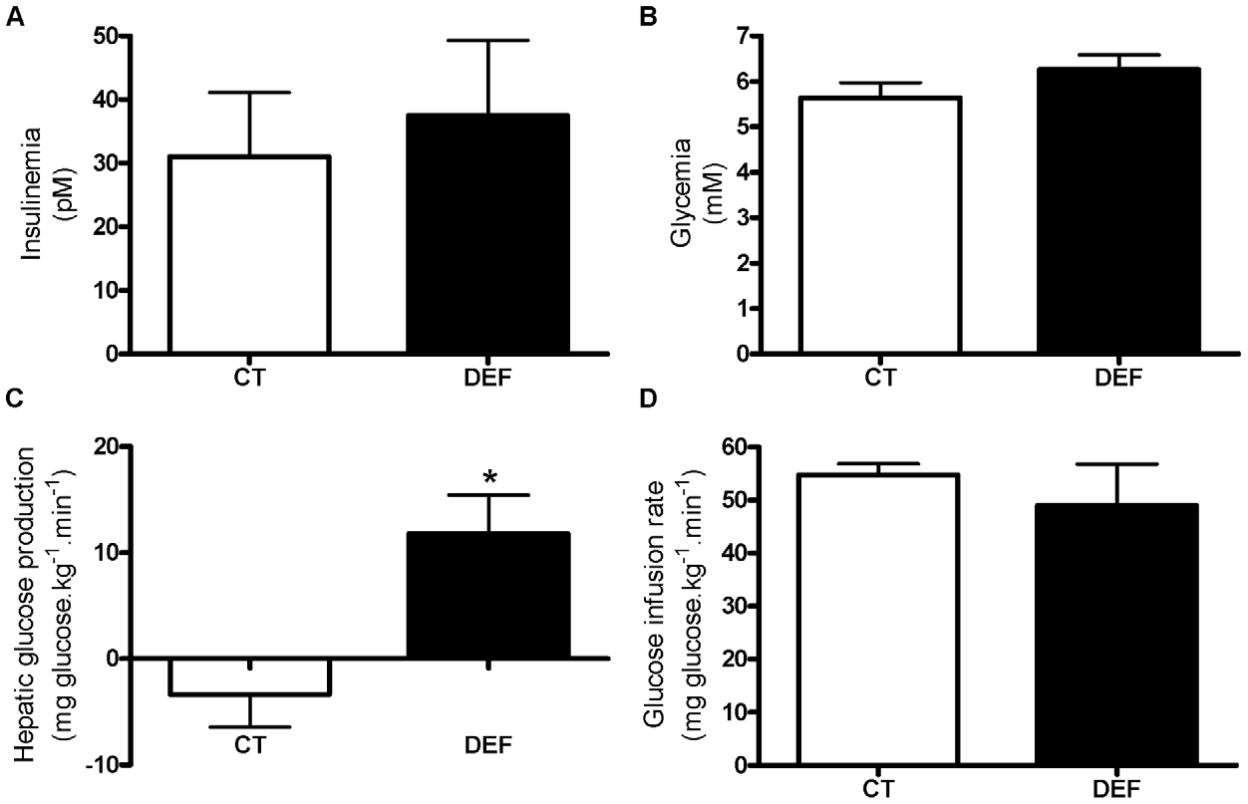
n-3 PUFA-depleted mice exhibited hepatic insulin resistance. Insulinemia (A) and glycemia (B) in fasted mice fed a control (CT; n = 6) or n-3 PUFA-depleted (DEF; n = 7) diet for 3 months. Hepatic glucose production (C) and glucose infusion rate (D) were measured during steady-state euglycemic-hyperinsulinemic clamp performed on fasted mice fed a control (CT; n = 4) or n-3 PUFA depleted (DEF; n = 6) diet for 3 months. Data are the mean ± SEM. ^*^: mean values significantly different (P<0.05, Student's *t*-test).

Insulin induces SREBP-1c activation by enhancing SREBP-1c transcription and proteolytic cleavage [20], [21]. As shown in Figure 5A and 5B, respectively, insulinemia and glycemia were similar between CT and DEF mice. Following euglycemic-hyperinsulinemic clamp, we found that DEF mice exhibited hepatic insulin resistance as shown by the higher hepatic glucose production upon insulin stimulation when compared to CT mice (Figure 5C). There were no differences in the glucose infusion rate between groups (Figure 5D). Thus, these data confirm the development of hepatic insulin resistance in DEF mice and argue against a role of insulin in SREBP-1c activation.

### n-3 PUFA depletion does not induce hepatic endoplasmic reticulum (ER) stress

**Figure 6:**
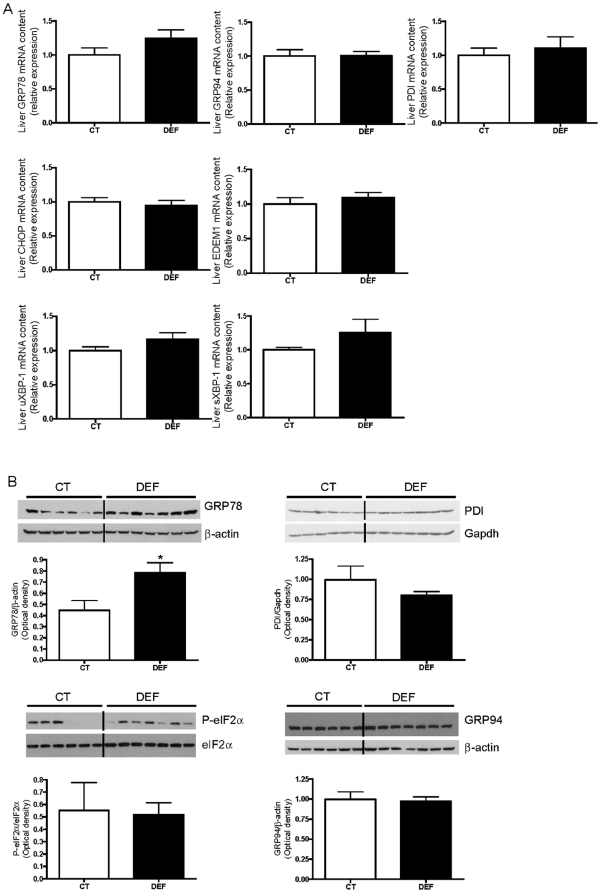
Absence of hepatic ER stress under n-3 PUFA depletion. Mice were fed a control (CT; n = 6) or n-3 PUFA-depleted (DEF; n = 7) diet for 3 months. The hepatic mRNA content of glucose-regulated protein 78 (GRP78), glucose-regulated protein 94 (GRP94), protein disulfide isomerise (PDI), C/EBP homologous protein (CHOP), ER degradation enhancer, mannosidase alpha-like 1 (EDEM1), unspliced (uXBP-1) and spliced (sXBP-1) X-box binding protein-1 is shown from fasted CT and DEF mice. The qPCR results were analysed according to the 2^−ΔΔ ct^ method and were normalised to RPL-19 mRNA (A). Immunoblot analysis of GRP78, PDI, GRP94 and total and phosphorylated eukaryotic translation initiation factor 2α (eIF2α) (B) in the livers from fasted CT and DEF mice. Quantification of immunoblots is shown below. Data are the mean ± SEM. ^*^: mean values significantly different (P<0.05, Student's *t*-test).

Kammoun *et al.* have demonstrated that the ER stress pathway induces SREBP-1c cleavage and expression independently from insulin [22]. Here the microarray analysis revealed an increase in the mRNA level of 3 markers of ER stress (Serp1, Sel1l and GRP94) in the livers of DEF mice compared to those of CT mice (Table S2), which was not confirmed by quantification of glucose-regulated protein 94 (GRP94) mRNA by qPCR (Figure 6).

We also analysed by western blot and qPCR the expression of several critical markers involved in the three axes of the unfolded protein response (UPR), namely inositol-requiring enzyme 1α (IRE1α), PKR-like ER kinase (PERK) and activating transcription factor 6 (ATF6). All of the results were similar between groups, except for glucose regulated protein 78 (GRP78) protein content which was higher in the liver of DEF mice despite no modification in its mRNA expression (Figure 6). These data suggest that ER stress is not involved in the SREBP-1c activation observed in n-3 PUFA-depleted mice.

### Increased Liver X receptor (LXR) activity occurs in the livers of n-3 PUFA-depleted mice

LXR, a transcription factor involved in the prevention of cholesterol toxicity, is able to stimulate fatty acid synthesis by increasing the expression of SREBP-1c [21]. Microarray analysis revealed that the mRNA content of several LXR target genes, i.e., PLTP, DEC1, Insig2 and Cyp7a1, were increased in the livers of DEF mice compared to those of CT mice. The increase of Insig2 and Cyp7a1 mRNA expression was confirmed by qPCR (Table S5). Moreover, the expression of Cyp7b1 and Enho, which is decreased by activated LXR, and Ncor1, an LXR inhibitor, was reduced in DEF mice compared to CT mice (Table S2). Based on these data, the TFactS analysis confirmed the activation of the LXR pathway (Table S4). In addition to the microarray expression profiling, we also measured the expression of the ATP-binding cassette transporter ABCG5, which reflects LXR activation, by qPCR. We found increased hepatic ABCG5 mRNA content in DEF mice compared to CT mice (Table S5). Altogether, these observations are in favour of an activation of the LXR pathway upon n-3 PUFA depletion.

## Discussion

Several papers suggest that decreased n-3/n-6 PUFA ratio in the diet is associated with changes in n-3/n-6 PUFA ratio in hepatic membrane phospholipids, on the one hand, and on the development of hepatic steatosis in humans, on the other hand [14]-[16]. Even if we can not exclude that metabolic changes of the liver (such as oxidative stress) may contribute to changes in hepatic fatty acid profile [15], it is conceivable that the imbalance dietary intake of PUFA plays a crucial role in the appearance of steatosis [14].

In a previous study, we have reported toxic steatosis in female mice fed with n-3 PUFA depleted- sucrose rich diet for two generations [18]. In this case, the hepatic morphological alterations were associated with a low expression of factors and enzymes involved in lipogenesis and an increase in the expression of those involved in fatty acid oxidation [18]. In the present study, we have created a model of nutritional n-3 PUFA depletion which did not provide any signs of hepatic toxicity that could compromise the interpretation of the metabolic data.

A targeted change in the lipid source of the diet allowed us to create a mouse model to explore the biochemical mechanisms underlying hepatic lipid accumulation under n-3 PUFA depletion. As previously shown in rats [23], 3 months of dietary n-3 PUFA depletion were sufficient to induce an altered fatty acid pattern in hepatic PLs, characterised by a large decrease in n-3 PUFA and a parallel increase in MUFA, without changing the total n-6 PUFA and saturated fatty acid levels. These changes were associated with hepatic accumulation of TG and esterified cholesterol, leading to a mixed macro- and microvesicular steatosis.

The microarray analysis revealed a reduced expression of PPARα and its target genes in the livers of DEF mice compared to those of CT mice. Moreover, PCLSs prepared from fasted DEF mice had a reduced conversion of palmitate into CO_2_ when compared to PCLSs from CT mice. Therefore, the decreased fatty acid oxidation observed in n-3 PUFA-depleted mice may contribute to hepatic steatosis. The in vitro studies using PCLS also revealed a higher capacity to synthesise fatty acids and TG from labelled precursors in the livers of DEF mice compared to those of CT mice. Microarray analysis confirmed a higher expression of all the enzymes involved in fatty acid synthesis in the livers of DEF mice compared to those of CT mice. The SCD-1 overexpression found in DEF mice could explain the increased MUFA content in hepatic PLs and, to a certain extent, the enhanced liver TG secretion observed under n-3 PUFA depletion, as an increased SCD-1 expression is associated with higher liver TG secretion in mice [24]. Numerous studies, both in vitro and in vivo, have demonstrated that n-3 PUFA supplementation decreases lipid synthesis [2] through inhibition of SREBP-1c gene expression and/or through the inhibition of the translocation of the active form of SREBP-1c in the nucleus [4]–[6], [25]. The ER protein Insig2a keeps SREBP-1c inactive at the ER membranes, and some studies have reported that Insig2a overexpression leads to a reduction of SREBP-1c activation [26]. In our study, we showed that SREBP-1c expression and the nuclear level of the SREBP-1 protein both increased despite a higher Insig2a expression.

Insulin is considered to be the classical driver of SREBP-1c activation, which largely explains carbohydrate-induced lipogenesis [20]. In DEF mice, we observed no changes in glycemia and insulinemia. Euglycemic-hyperinsulinemic clamp revealed a lower inhibitory effect of insulin on hepatic glucose production in DEF mice compared to CT mice. These results demonstrate that n-3 PUFA depletion promotes hepatic insulin resistance. Therefore, SREBP-1c activation may be driven by an insulin-independent pathway.

A recent study has suggested that the increased lipogenesis observed in an insulin-resistant state could result from ER stress [22]. To maintain ER function when the secretory pathway is compromised, cells have developed an adaptive mechanism called UPR [27], [28]. Activation of the UPR pathway occurs together with activation of SREBP-1c in the livers of obese *ob/ob* mice and in nutritional models of steatosis such as hyperhomocysteinaemia or alcohol-fed rodents [22], [29], [30]. IRE1α, ATF6 and PERK are three proximal sensors of ER stress [27], [28]. Some studies have pointed out the importance of IRE1α/X-box binding protein-1 (XBP-1) and PERK pathways activation for the stimulation of lipogenesis in genetic deletion models [31]. Here we did not observe any modifications in the expression and/or the protein content of PDI, GRP94, EDEM1, unspliced XBP-1, spliced XBP-1 or CHOP, all involved in the 3 branches of the UPR. Moreover, eukaryotic translation initiation factor 2α (eIF2α) phosphorylation was similar between groups. Of note, ER stress is also associated with lower Apo B and hepatic TG secretion [31]. Here we found that TG secretion was increased upon n-3 PUFA depletion. Foufelle's group has demonstrated that GRP78 overexpression in the liver of *ob/ob* mice could inhibit SREBP-1c proteolytic cleavage [22]. Curiously, we observed an increased SREBP-1c activation in the liver of DEF mice despite a higher hepatic GRP78 protein content. Altogether, these results provide evidence against the involvement of ER stress in the hepatic lipid metabolism alterations occurring in n-3 PUFA-depleted mice.

LXR is another nuclear factor involved in the regulation of fatty acid synthesis [32]; it is required for the full induction of the SREBP-1c promoter by insulin [33], [34]. Microarray analysis and qPCR quantification of LXR target gene expression suggested increased activity of LXR in the livers of DEF mice compared to those of CT mice. Additionally, DEF mice exhibited reduced expression of Ncor1, a major LXR corepressor [35]. The increased LXR activity observed here could be due to the decrease in tissue n-3 PUFA content. Indeed, studies have reported that n-3 PUFA can inhibit the natural binding of oxysterols, the endogenous LXR ligands, to the receptor, leading to decreased SREBP-1c gene transcription [36]–[39]. Other studies have reported that n-6 PUFA, especially arachidonic acid, can also inhibit LXR activity [40]. However, this does not seem to be the case here, as total n-6 PUFA content was similar and arachidonic acid (C20:4) content was even higher in hepatic PLs of DEF mice than in those of CT mice.

Higher cholesterol content has also been observed in the livers of DEF mice, and it is associated with an increased expression of enzymes involved in cholesterol synthesis and an activation of SREBP-2. Some authors have suggested that cholesterol overloading leads to LXR activation through the production of 27-hydroxycholesterol [41] or of 24(*S*),25-epoxycholesterol, which is derived from a shunt in the cholesterol biosynthetic pathway [42]. LXR activation is also dependent on the expression of Cyp7b1, an enzyme involved in the alternative bile acid synthesis pathway that degrades oxysterols [43], [44]. Interestingly, expression of Cyp7b1 was lower in the livers of DEF mice than in those of CT mice. Thus, our data suggest that n-3 PUFA depletion promotes the production of endogenous LXR ligands and that this increase could in turn enhance LXR activity in the liver. The increased LXR activity might also contribute to the higher hepatic TG secretion observed in n-3 PUFA-depleted mice, as LXR ligands have been shown to induce Apo B-dependent TG secretion by increasing PLTP expression in mice [45].

Of note, we found that DEF mice exhibited a higher hepatic content of 2-AG than CT mice, which could result from the lower expression of monoacylglycerol lipase (MGL). This enzyme is indeed mostly involved in 2-AG catabolism (Table S2). It has been shown that n-3 PUFA depletion elevates and n-3 PUFA supplementation reduces the 2-AG level in the brain of mice [46]. A few papers suggest that the endocannabinoids, 2-AG being classified among them, are bioactive lipids involved in the regulation of the hepatic lipid homeostasis [47]. Indeed, it has been reported that CB_1_ endocannabinoid receptor activation in mice induces SREBP-1c and its target lipogenic enzymes such as FAS in the liver and in the adipose tissue [48], [49]. In hepatic stellate cells an increased 2-AG level is associated with increased SREBP-1c expression and lipogenesis [50]. Therefore, we speculate that the increase in 2-AG content might also influence the higher SREBP-1c expression occurring in DEF mice.

Carbohydrate-responsive element-binding protein (Chrebp) is another key transcription factor involved in the regulation of lipogenic gene expression [51]. It has been reported that n-3 PUFA could inhibit the lipogenesis by inhibiting SREBP-1c pathway, but also by altering Chrebp translocation to the nucleus [52], [53]. Our data revealed no difference between CT and DEF mice in hepatic Chrebp mRNA content. However, we found in the liver of DEF mice an increased in L-PK expression (Table S2, S5), which constitute on target gene of Chrebp. Therefore, we can not rule out a potential implication of Chrebp in the inhibitory effect of n-3 PUFA on lipogenesis, even if its contribution remains less clear than the one of SREBP-1c.

**Figure 7:**
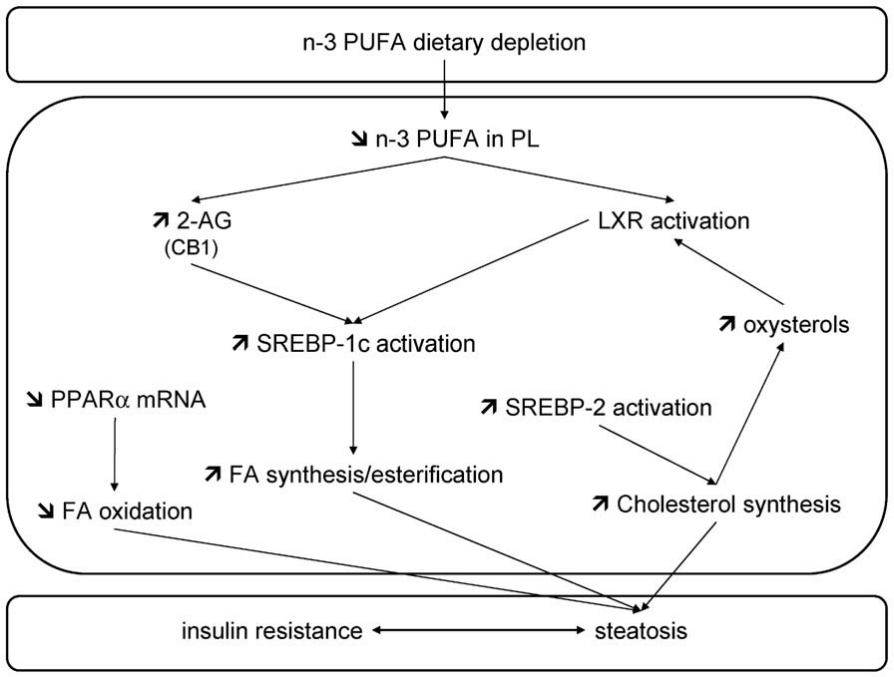
Proposed metabolic pathways involved in fatty acid and cholesterol accumulation in the livers of n-3 PUFA depleted mice. The reduction of fatty acid oxidation and the induction of lipogenesis both contribute to the accumulation of lipids in the livers of n-3 PUFA-depleted mice. The reduced fatty acid degradation could result from the inhibition of the PPARα pathway. De novo lipogenesis is promoted through SREBP-1c activation. Data suggest that SREBP-1c activation could result from both increased hepatic 2-AG content and LXR activation. The lower n-3 PUFA content in hepatic phospholipids can lead to higher 2-AG production, which could therefore stimulate CB1 and then SREBP-1c expression. Both hepatic n-3 PUFA depletion and higher cholesterol content contribute to LXR activation. Increased SREBP-2 activation, occurring by an unknown mechanism, could play a role in the increased cholesterol synthesis observed in the n-3 PUFA-depleted livers.

In conclusion the metabolic characteristics observed in our model of n-3 PUFA depletion are opposite to the ones occurring upon n-3 PUFA supplementation [2]. The consumption of a diet containing low levels of n-3 PUFA for 3 months was sufficient to induce hepatic n-3 PUFA depletion in PLs, steatosis (despite the maintenance of VLDL secretion process) and insulin resistance. Decreased fatty acid oxidation and increased TG and cholesterol synthesis both contributed to lipid accumulation. The activation of SREBP-1c-related pathways was particularly interesting, as it occurred in a hepatic insulin-resistant state and independently of ER stress, and it is consistent with increased LXR activity and a higher endocannabinoid ligand level (2-AG) (Figure 7). Even if we may not directly extrapolate our experimental data obtained in mice to human health, it is interesting to note, in accordance with our experimental data, that human biopsies of non-alcoholic fatty liver disease patients are characterized by an increased expression of SREBP1c and decreased expression of PPARα and this was associated with n-3 PUFA depletion [54]. Our results provide new insights into the effect of nutritional disequilibrium at the expense of n-3 PUFA on the occurrence of steatosis.

## Materials and Methods

### Ethics statement

The animal experiments were reviewed and approved by the local ethic committee; the agreement of the animal experiments performed in this study was given by the ethical committee for animal care of the Health Sector of the Université catholique de Louvain, under the supervision of prof. P. Gianello P et JP Dehoux under the specific number ULC/MD/2007/003. Housing conditions were as specified by the Belgian Law of 14 November, 1993 on the protection of laboratory animals (agreement n° LA 1230314).

### Animals and diets

Male C57Bl/6J mice (9 weeks old; Charles River, Brussels, Belgium) were housed in groups of 4 mice per cage at 22°C in a 12 h light/dark cycle and given free access to diet and water.

After an acclimatisation period of 1 week, mice were fed a control (CT) (D08041805, Research Diets, New Brunswick, USA) or an n-3 PUFA-depleted diet (DEF) (D08041806, Research Diets, New Brunswick, USA) for 3 months ad libitum. The n-3 PUFA depletion was induced by replacing the soybean oil with sunflower oil; all other nutrients including MUFA and saturated fatty acid content were similar to those of the CT diet (Table S6, S7).

At the end of the study period, mice were anaesthetised (ketamine/xylazine i.p., 100 and 10 mg/kg of body weight, respectively) after a 6 h fasting period (CT, n = 6; DEF, n = 7). All biochemical measurements, qPCR, western blot and microarray analysis were performed on these fasted mice. For comparison with the results obtained in the fasted state, microarrays were also run using fed mice (CT, n = 4; DEF, n = 7). In addition, other mouse experiments were conducted for in vivo measurement of hepatic TG secretion, euglycemic-hyperinsulinemic clamp studies and PCLS assessment of hepatic fatty acid oxidation and TG synthesis. Except for the latter, these studies were carried out in fasted mice.

### Blood biochemical analysis

Blood glucose was determined before anaesthesia with a glucose meter (Roche diagnostic) on 3.5 µl of blood collected from the tip of the tail vein. Vena cava blood samples were collected in EDTA tubes. After centrifugation (3 min at 13000 *g*), plasma was stored at −80°C until analysis. Insulin was measured in 5 µl of plasma using an ELISA kit (Mercodia, Upssala, Sweden).

### Liver histological analysis

For the detection of neutral lipids, frozen sections obtained from a fraction of the main liver lobe mounted in embedding medium (Tissue-tek, Sakura) were sliced and stained with the oil red O, using 0.5% oil red O dissolved in propylene glycol for 10 min at 60°C. The sliced sections were then counterstained.

### Tissue biochemical analysis

The remaining liver tissue after histological sampling was immediately clamped in liquid N_2_ and kept at −80°C until analysis. Fatty acid content was determined in tissue PLs of a pool of 4 mice per group as reported before [55]. For hepatic lipid content measurement, lipids were extracted with chloroform-methanol (2∶1) according to Folch et al. [56]. TG, total and free cholesterol concentrations were measured using kits (Diasys Diagnostic and Systems, Holzheim, Germany) coupling enzymatic reaction and spectrophotometric detection of reaction end-products. Esterified cholesterol was calculated as the subtraction between total cholesterol and free cholesterol.

### Measurement of endocannabinoids levels

Liver tissues were homogenized in CHCl_3_ (10 ml) and 200 pmol of each deuterated standard (d-AEA, d-OEA, d-PEA, d-SEA, d-2-AG) were added. MeOH (5 ml) and H_2_O (2.5 ml) were then added and the lipids extracted by vigorous mixing. Following centrifugation, the organic layer was recovered, dried under a stream of N_2_ and purified by solid phase extraction using silica and eluted with EtOAc-Acetone (1∶1) [57], [58]. The resulting lipid fraction was analyzed by HPLC-MS using a LTQ Orbitrap mass spectrometer (ThermoFisher Scientifc) coupled to an Accela HPLC system (ThermoFisher Scientific). Analytes separation was achieved using a C-18 Supelguard pre-column and a Supelcosil LC-18 column (3 µM, 4×150 mm) (both from Sigma-Aldrich). Mobile phase A and B were composed of MeOH-H_2_O-acetic acid 75∶25∶0.1 (v/v/v) and MeOH-acetic acid 100∶0.1 (v/v), respectively. The gradient (0.5 ml/min) was designed as follows: from 100% A to 100% B in 15 min, followed by 10 min at 100% B and subsequent re-equilibration at 100% A. MS analysis in the positive mode was performed by APCI ionization with the capillary and APCI vaporizer temperatures set at 250°C and 400°C, respectively [49]. Endocannabinoids were quantified by isotope dilution using its deuterated standard (showing identical retention times). The calibration curves were generated as described [57] and the data normalized by tissue sample weight.

### cDNA microarray analysis

Total RNA was isolated from liver tissue of fasted and fed mice using the TriPure reagent (Roche, Basel, Switzerland). RNA quality was checked using Bioanalyzer (Agilent). Equal amounts RNA from each mice (n = 4 to 7 mice per group) were pooled within each group. Microarray experiments were performed as described [59], [60]. Double-stranded cDNA was synthesized from total RNA using the One-cycle cDNA synthesis kit (Affymetrix, Santa Clara, USA). Biotin-labeled cRNA was synthesized using GeneChip IVT labelling kit (Affymetrix, Santa Clara, USA). After fragmentation, cRNA was hybridized to mouse genome 430 2.0 array (Affymetrix, Santa Clara, USA). The MAS5 algorithm was run using GCOS® Affymetrix software as follows: the scaling factor using all probe sets was set to 100, the normalize factor was set to 1 and the baseline comparison was done on the CT diet hybridization samples. Probe sets that were ‘absent’ or ‘NC’ in the four conditions were eliminated. Then, only genes marked as significantly ‘Increased’ or ‘Decreased’ upon n-3 PUFA depletion in both fasted and fed mice were considered as the regulated gene list.

The regulated gene list was submitted to the DAVID web server for functional enrichment analysis against ontologies such as: Gene Ontology (GO), Kegg pathways and SwissProt PIR (SP-PIR) keywords. We considered a P-value threshold of 0.05 as significant.

TFactS predicts transcription factor regulation form gene lists based on the comparison with a database of experimentally-validated target genes. The regulated gene list was submitted to TFactS (www.tfacts.org). Only transcription factors with at least 10 target genes in signature were analysed. We considered an E-value <0.05 as significant. All data are MIAME compliant and the raw data has been deposited in a MIAME compliant database; the GEO database (accession number GSE26986).

### Real-time quantitative PCR

cDNA was prepared by reverse transcription of 1 µg total RNA using the Kit Reverse transcription System (Promega, Leiden, The Netherlands). Real-time qPCRs were performed with a StepOnePlusTM instrument and software (Applied Biosystems, Foster City, CA, USA) using Mesa Fast qPCRTM (Eurogentec, Seraing, Belgium) as described [18]. Primers and gene details are summarized in Table S8.

### SDS/PAGE and immunoblotting

For total lysates, approximately 30 mg of frozen liver were homogenized with TissueLyser II (Qiagen) in RIPA buffer (25 mM Tris-HCl pH 7.6, 150 mM NaCl, 1% NP-40, 1% deoxycholic acid, 0.1% sodium dodecyl sulphate) supplemented with a cocktail of protease inhibitors (P8340, Sigma-Aldrich, Saint Louis, USA) and of phosphatase inhibitors (Calbiochem, Nottingham, UK). The homogenates were then centrifuged for 20 min at 13000 *g*. Cytoplasmic and nuclear proteins were extracted following manufacturer's instruction (NE-PER®, Thermo Scientific, Waltham, USA) from 50 mg of liver tissue. Equal amount proteins were separated by SDS/PAGE and transferred to nitrocellulose membrane. The membranes were incubated overnight at 4°C with the following antibodies diluted in tris-buffered saline tween-20 containing 1% non fat dry milk: GRP78 (1∶1000), GRP94 (1∶1000), PDI (protein disulfide isomerise, 1∶1000), total eIF2α (1∶500), SREBP-2 (1∶200) and SREBP-1 (1∶1000). P-eIF2α (1∶1000) was diluted in tris-buffered saline tween-20 without non fat dry milk. All antibodies were purchased from Abcam except GRP94, GRP78 and PDI (Cell signalling, Danvers, USA). GAPDH and β-actin (Abcam, Cambridge, UK) were used as loading control. The films were quantified using ImageJ software.

### Hepatic fatty acids synthesis, esterification and oxidation

For fatty acid synthesis and esterification, precision-cut liver slices (PCLS) were prepared with a Krumdieck Tissue Slicer from CT (n = 7) and DEF (n = 8) fed mice according to a procedure previously described [61]. PCLS were preincubated 30 minutes in ice cold Waymouth medium supplemented with insulin (0.1 µM), penicillin/streptomycin (1%) and bovine serum albumin (0.3%). After 30 minutes of preincubation, PCLS were transferred into Waymouth medium (supplemented with 0.1 µM insulin, 1% penicillin/streptomycin and 0.1% bovine serum albumin) containing 2 mM [^14^C]-acetate (0.2 mCi/mmol) or 0.2 mM [^14^C]-palmitate (0.2 mCi/mmol) and incubated for 3 h. Incubations were carried out at 37°C under an atmosphere of O_2_/CO_2_ (95∶5%) in a shaking water-bath. PCLS were sonicated in 0.4 ml NaCl (0.9%) before the addition of 2.5 ml of isopropanol/heptane (4∶1) for TG extraction. After 1 h of incubation at room temperature, 1.5 ml of heptane and 1 ml of water were added to the samples. For slices incubated with palmitate, the organic phase was rinsed two times with NaOH 1N/water/ethanol (5∶45∶50). TG collected from the upper organic phase was counted in 10 ml scintillation fluid (Ultima Gold) in a beta Wallac-1410 counter.

For fatty acid oxidation, PCLS were prepared from CT (n = 8) and DEF (n = 6) fasted mice. After 30 minutes of preincubation, PCLS were incubated in Waymouth medium (supplemented with 0.1 µM insulin, 1% penicillin/streptomycin and 0.1% bovine serum albumin) containing 0.2 mM [^14^C]-palmitate (0.4 mCi/mmol) for 3 h at 37°C in a shaking water-bath. Flasks were gassed with O_2_/CO_2_ (95∶5) and sealed with rubber caps equipped with suspended plastic center wells, containing 400 µl of NaOH 10%. Incubations were terminated by addition of 1 ml of HCLO_4_ 5%. Control flasks were acidified immediately prior to addition of liver slices. To trap the ^14^CO_2_, an additional 60 min of shaking was allowed. To determine the amount of produced CO_2_, all the NaOH was counted in 10 ml scintillation fluid.

### Hepatic triglycerides secretion

Following a 4 h fasting period, CT (n = 4) and DEF (n = 6) mice were injected with a 10% solution of Tyloxapol (0.5 mg of Tyloxapol/g body weight) (T0307-5G, Sigma-Aldrich, Saint Louis, USA) through the tail vein to inhibit TG degradation by lipoprotein lipase. Plasma samples were collected through the tail vein before the injection (0 min) and 30, 60, 90 and 120 minutes after Tyloxapol injection. TG in plasma was measured using Diasys Diagnostic and Systems kit (Holzheim, Germany).

### Euglycemic-Hyperinsulinemic clamp study

Mice were anesthetized with isoflurane (Abbott, Rungis, France) and a catheter was indwelled into the femoral vein as previously described [62]. Then, mice were housed individually and were allowed to recover for 10 days. Hepatic Insulin sensitivity was assessed by the euglycemic-hyperinsulinemic clamp as described [62], [63]. Mice (CT n = 4; DEF n = 6) fasted for 5 h, were infused with human insulin (Actrapid) at a rate of 2.5 mU · kg−1 · min−1 for 3 h and d-(^3^H)3-glucose (Perkin Elmer, Boston, MA) was infused at rate of 0.33 µCi · min−1 to ensure a detectable plasma d-(^3^H)3-glucose enrichment. Throughout the infusion, tail blood glycemia was assessed every 10 min using a blood glucose meter (Accu-chek, Aviva, Roche). Euglycemia was maintained by periodically adjusting a variable infusion of 20% (wt/vol) glucose. When the steady-state was obtained, plasma glucose concentrations and d-(^3^H)3-glucose specific activity were determined in 5 µl of blood sampled from the tip of the tail vein every 10 min during the last hour of the infusion.

### Statistical analysis

Results are presented as mean ± SEM. Statistical significance was assessed by a Student *t*-test or Two-way ANOVA using GraphPad Prism version 4.00 for Windows. P<0.05 was considered as statistically significant.

## Supporting Information

Table S1 **Endocannabinoids content in the liver of CT and DEF mice.** 2-arachidonoylglycerol (nmol/g liver tissue), *N*-arachidonoylethanolamine (pmol/g liver tissue), *N*-palmitoylethanolamine (pmol/g liver tissue), *N*-stearoylethanolamine (pmol/g liver tissue) and *N*-oleoylethanolamine (pmol/g liver tissue) content in the liver of fasted mice fed with a control (CT; n  =  6) or n-3 PUFA depleted diet (DEF; n  =  7) for 3 months. Data are the mean ± SEM. ^*^: mean values significantly different (P<0.05, Student *t*-test)
(DOC)

Table S2 **Genes significantly modulated in the liver of n-3 PUFA depleted mice (DEF) compared to control mice (CT).** Genes significantly up (positive values) or downregulated (negative values) in the liver of mice fed with an n-3 PUFA depleted (DEF) diet for three months compared to mice fed with a control (CT) diet in the fasted and fed state. Results are obtained from microarray analyses and are expressed as Log_2_ of the fold changes in DEF versus CT values. NC  =  not significantly changed between CT and DEF mice.
(DOC)

Table S3 **Pathway and gene ontology analysis using DAVID.** The consensus list of regulated genes was submitted to DAVID. Terms that were significantly represented (corrected P-values and FDR <0.05) are shown. Redundant terms were removed.
(DOC)

Table S4 **Transcription factor predictions by TFactS.** The list of genes changed in n-3 PUFA depleted mice compared to control mice (in the fed and starved conditions) was submitted to TFactS (sign-less) using default settings. Transcription factors that are significantly regulated with P-value, E-value, Q-value, FDR and random control <5% are shown. The regulation type (activation or inhibition) is indicated if it was found significant (p<0.05) by TFactS (sign-sensitive). HNF4 is not present in the latter database. Only transcription factors with a minimum of 10 target genes in signature were analysed.
(DOC)

Table S5 **qPCR confirmation of genes modulated in the liver of n-3 PUFA depleted mice (DEF) compared to control mice (CT).** mRNA content of sterol-regulatory-element-binding protein-1c (SREBP-1c), fatty acid synthase (FAS), stearoyl-CoA desaturase-1 (SCD-1), ATP-binding cassette transporters G5 (ABCG5), insulin induced gene 2a (Insig2a), carbohydrate-responsive element-binding protein (Chrebp), liver-pyruvate kinase (L-PK), cytochrome P450, family 7, subfamily A, polypeptide 1, (cyp7a1) and 3-hydroxy-3-methyl-glutaryl-CoA reductase (HMGCoAr) in the liver of mice fed a control (CT; n  =  6) or n-3 PUFA depleted diet (DEF; n  =  7) for three months. Results were analysed according to the 2^−ΔΔ ct^ method and were normalised to RPL-19 mRNA. Data are the mean ± SEM. ^*^: mean values significantly different (P<0.05, Student *t*-test)
(DOC)

Table S6 **Composition of control (CT) and n-3 PUFA depleted (DEF) diet.** Formulated by Research Diets. Parenthetical numbers indicate the manufacturer's diet number.
(DOC)

Table S7 **Fatty acid composition of soybean and sunflower oil.** Fatty acid composition of soybean and sunflower oil used for the CT and DEF diet respectively.
(DOC)

Table S8 **Primers sequences used for real-time quantitative PCR.** RPL19; ribosomal protein L19, FAS; fatty acid synthase, SREBP-1c; sterol-regulatory-element-binding protein-1c, Chrebp; carbohydrate-responsive element-binding protein, L-PK; liver-pyruvate kinase, SCD-1; stearoyl-CoA desaturase-1, ATP-binding cassette transporters G5; ABCG5, cyp7a1; cytochrome P450, family 7, subfamily A, polypeptide 1, Insig2a; insulin induced gene 2a, HMGCoAr; 3-hydroxy-3-methyl-glutaryl-CoA reductase, EDEM1; ER degradation enhancer, mannosidase alpha-like 1, spliced (s) and unspliced (u) XBP-1; X-box binding protein 1, GRP78; glucose-regulated protein 78, GRP94; glucose-regulated protein 94, CHOP; CCAAT/enhancer binding protein homologous protein, PDI; protein disulfide isomerise.
(DOC)
