# Supplementary material for: Hepatic n-3 Polyunsaturated Fatty Acid Depletion Promotes Steatosis and Insulin Resistance in Mice: Genomic Analysis of Cellular Targets
Source: PLoS One. 2011 Aug 10;6(8):e23365. doi: 10.1371/journal.pone.0023365 (PMC3154437; doi:10.1371/journal.pone.0023365)
Supplement: Table S1 — Endocannabinoids content in the liver of CT and DEF mice. 2-arachidonoylglycerol (nmol/g liver tissue), N-arachidonoylethanolamine (pmol/g liver tissue), N-palmitoylethanolamine (pmol/g liver tissue), N-stearoylethanolamine (pmol/g liver tissue) and N-oleoylethanolamine (pmol/g liver tissue) content in the liver of fasted mice fed with a control (CT; n = 6) or n-3 PUFA depleted diet (DEF; n = 7) for 3 months. Data are the mean ± SEM. *: mean values significantly different (P<0.05, Student t-test) (DOC) [file pone.0023365.s001.doc]

**Table S1**: Endocannabinoids content in the liver of CT and DEF mice

|  | CT | DEF |
| --- | --- | --- |
| 2-arachidonoylglycerol (nmol/g liver tissue) | 0.7 ± 0.1 | 1.0 ± 0.1* |
| *N*-arachidonoylethanolamine (pmol/g liver tissue) | 18.5 ± 2.2 | 18.6 ± 1.3 |
| *N*-palmitoylethanolamine (pmol/g liver tissue) | 105.5 ± 14.2 | 126.0 ± 18.3 |
| *N*-stearoylethanolamine (pmol/g liver tissue) | 48.9 ± 10.2 | 53.1 ± 11.4 |
| *N*-oleoylethanolamine (pmol/g liver tissue) | 55.7 ± 10.1 | 90.2 ± 15.5 |

2-arachidonoylglycerol (nmol/g liver tissue), *N*-arachidonoylethanolamine (pmol/g liver tissue), *N*-palmitoylethanolamine (pmol/g liver tissue), *N*-stearoylethanolamine (pmol/g liver tissue) and *N*-oleoylethanolamine (pmol/g liver tissue) content in the liver of fasted mice fed with a control (CT; n = 6) or n-3 PUFA depleted diet (DEF; n = 7) for 3 months.

Data are the mean ± SEM

^*^: mean values significantly different (P<0.05, Student *t*-test)
