# Supplementary material for: Hepatic n-3 Polyunsaturated Fatty Acid Depletion Promotes Steatosis and Insulin Resistance in Mice: Genomic Analysis of Cellular Targets
Source: PLoS One. 2011 Aug 10;6(8):e23365. doi: 10.1371/journal.pone.0023365 (PMC3154437; doi:10.1371/journal.pone.0023365)
Supplement: Table S2 — Genes significantly modulated in the liver of n-3 PUFA depleted mice (DEF) compared to control mice (CT). Genes significantly up (positive values) or downregulated (negative values) in the liver of mice fed with an n-3 PUFA depleted (DEF) diet for three months compared to mice fed with a control (CT) diet in the fasted and fed state. Results are obtained from microarray analyses and are expressed as Log2 of the fold changes in DEF versus CT values. NC = not significantly changed between CT and DEF mice. (DOC) [file pone.0023365.s002.doc]

**Table S2.** Genes significantly modulated in the liver of n-3 PUFA depleted mice (DEF) compared to control mice (CT)

| Accession number | Description | Fasted state Fold (Log_2_) | Fed state Fold (Log_2_) |
| --- | --- | --- | --- |
| NM_009127.1 | Stearoyl-Coenzyme A desaturase 1 (SCD-1) | 1.4 | 0.7 |
| AF332052.1 | ATP citrate lyase (Acly) | 1.1 | 0.8 |
| BI456232 | ATP citrate lyase (Acly) | 0.6 | 0.6 |
| BB276877 | ATP citrate lyase (Acly) | 0.4 | 0.5 |
| BI247584 | Farnesyl diphosphate synthetase (Fdps) | 1.1 | 0.6 |
| AK006387.1 | Malic enzyme 1 (Me1) | 1.1 | 0.5 |
| NM_013631.1 | Liver-pyruvate kinase (L-PK) | 1.0 | NC |
| BB667651 | Liver-pyruvate kinase (L-PK) | 0.4 | NC |
| AF127033.1 | Fatty acid synthase (FAS) | 0.9 | 1 |
| NM_011125.1 | Phospholipid transfer protein (PLTP) | 0.9 | 0.8 |
| AI591480 | Phospholipid transfer protein (PLTP) | 0.6 | 0.6 |
| NM_007474.1 | Aquaporin 8 (Aqp8) | 0.8 | 0.5 |
| BC022940.1 | Acetyl-Coenzyme A carboxylase beta (ACCβ) | 0.7 | 1 |
| BE650741 | Acetyl-Coenzyme A carboxylase alpha (ACCα) | 0.7 | 0.6 |
| BG921646 | Acetyl-Coenzyme A carboxylase alpha (ACCα) | 0.6 | 0.6 |
| BI713896 | Phosphomevalonate kinase (Pmvk) | 0.7 | 0.4 |
| NM_130450.1 | ELOVL family member 6 (Elovl6) | 0.6 | 0.7 |
| AF480860.1 | ELOVL family member 6 (Elovl6) | 0.3 | 0.6 |
| NM_019699.1 | Fatty acid desaturase 2 (FADS2) | 0.6 | 0.6 |
| BC019945.1 | NAD(P) dependent steroid dehydrogenase-like (Nsdhl) | 0.6 | 0.5 |
| AI326423 | Sterol regulatory element binding protein-1 (SREBP-1) | 0.5 | 0.8 |
| NM_007856.1 | 7-dehydrocholesterol reductase (Dhcr7) | 0.5 | 0.6 |
| BC011139.1 | Glucokinase (GK) | 0.5 | 0.5 |
| BI691849 | Sel-1 suppressor of lin-12-like (Sel1l) | 0.4 | 0.4 |
| NM_134255.1 | ELOVL family member 5 (Elovl5) | 0.4 | 0.3 |
| AB016248.1 | Sterol-C5-desaturase (Sc5d) | 0.4 | 0.3 |
| BB667338 | Cytochrome P450, family 7, subfamily a, polypeptide 1 (Cyp7a1) | 0.4 | 0.3 |
| BF658806 | Stress-associated endoplasmic reticulum protein 1 (Serp1) | 0.4 | 0.3 |
| AK014742.1 | Lanosterol synthase (Lss) | 0.3 | 0.7 |
| AV148646 | Acetyl-Coenzyme A acetyltransferase 2 (ACAT2) | 0.3 | 0.6 |
| BE995678 | Heat shock protein 90 beta, member 1 (GRP94) | 0.3 | 0.4 |
| BB705380 | 3-hydroxy-3-methylglutaryl-Coenzyme A synthase 1 (HMGCoAs) | 0.3 | 0.4 |
| NM_133748.1 | Insulin induced gene 2 (Insig2) | 0.3 | 0.3 |
| AV257512 | Insulin induced gene 2 (Insig2) | 0.5 | 0.3 |
| BC026831.1 | Fatty acid desaturase 1 (Fads1) | 0.3 | 0.3 |
| NM_011498.1 | Basic helix-loop-helix family, member e40 (DEC1) | 0.2 | 0.4 |
| AK006541.1 | Acyl-CoA synthetase long-chain family member 5 (Acsl5) | 0.2 | 0.3 |
| AI785548 | Apolipoprotein B (Apo B) | 0.2 | -0.9 |
| NM_007825.1 | Cytochrome P450, family 7, subfamily b, polypeptide 1 (Cyp7b1) | -0.2 | -0.3 |
| AV282267 | Peroxisome proliferator activated receptor alpha (PPARα) | -0.3 | -0.2 |
| AK017345.1 | Pantothenate kinase 1 (Pank1) | -0.3 | -0.4 |
| BI411560 | Monoacylglycerol lipase (MGL) | -0.4 | 0 |
| AI327006 | Cytochrome P450, family 4, subfamily a, polypeptide 14 (Cyp4a14) | -0.4 | -0.5 |
| NM_011396.1 | Solute carrier family 22 (organic cation transporter), member 5 (Octn2) | -0.5 | -0.5 |
| AW555798 | Nuclear receptor co-repressor 1 (Ncor1) | -0.5 | -0.6 |
| BC013476.1 | Cytochrome P450, family 4, subfamily a, polypeptide 31 (Cyp4a10) | -0.5 | -1 |
| BB772205 | Energy homeostasis associated (Enho) | -1 | -1.5 |

Genes significantly up (positive values) or downregulated (negative values) in the liver of mice fed with an n-3 PUFA depleted (DEF) diet for three months compared to mice fed with a control (CT) diet in the fasted and fed state. Results are obtained from microarray analyses and are expressed as Log_2_ of the fold changes in DEF versus CT values.

NC = not significantly changed between CT and DEF mice.
