# Supplementary material for: Hepatic n-3 Polyunsaturated Fatty Acid Depletion Promotes Steatosis and Insulin Resistance in Mice: Genomic Analysis of Cellular Targets
Source: PLoS One. 2011 Aug 10;6(8):e23365. doi: 10.1371/journal.pone.0023365 (PMC3154437; doi:10.1371/journal.pone.0023365)
Supplement: Table S3 — Pathway and gene ontology analysis using DAVID. The consensus list of regulated genes was submitted to DAVID. Terms that were significantly represented (corrected P-values and FDR <0.05) are shown. Redundant terms were removed. (DOC) [file pone.0023365.s003.doc]

**Table S3.** Pathway and gene ontology analysis using DAVID

| Category | Term | Count | Pop Hits | Fold Enrichment |
| --- | --- | --- | --- | --- |
| GO | Lipid biosynthetic process | 21 | 256 | 11.9 |
| GO | Steroid metabolic process | 15 | 155 | 14.1 |
| GO | Endoplasmic reticulum | 24 | 744 | 4.9 |
| GO | Alcohol metabolic process | 16 | 282 | 8.3 |
| GO | Cholesterol metabolic process | 10 | 70 | 20.8 |
| KEGG | PPAR signalling pathway | 10 | 73 | 10.3 |
| SP_PIR | Fatty acid biosynthesis | 7 | 41 | 24.3 |
| SP_PIR | Muscle protein | 7 | 44 | 22.7 |
| GO | Coenzyme metabolic process | 11 | 188 | 8.5 |
| KEGG | Polyunsaturated fatty avid biosynthesis | 6 | 17 | 26.7 |
| GO | Fatty acid biosynthetic process | 7 | 69 | 14.8 |
| KEGG | Biosynthesis of steroids | 6 | 23 | 19.7 |

The consensus list of regulated genes was submitted to DAVID. Terms that were significantly represented (corrected p-values and FDR <0.05) are shown. Redundant terms were removed.
