# Supplementary material for: Hepatic n-3 Polyunsaturated Fatty Acid Depletion Promotes Steatosis and Insulin Resistance in Mice: Genomic Analysis of Cellular Targets
Source: PLoS One. 2011 Aug 10;6(8):e23365. doi: 10.1371/journal.pone.0023365 (PMC3154437; doi:10.1371/journal.pone.0023365)
Supplement: Table S4 — Transcription factor predictions by TFactS. The list of genes changed in n-3 PUFA depleted mice compared to control mice (in the fed and starved conditions) was submitted to TFactS (sign-less) using default settings. Transcription factors that are significantly regulated with P-value, E-value, Q-value, FDR and random control <5% are shown. The regulation type (activation or inhibition) is indicated if it was found significant (p<0.05) by TFactS (sign-sensitive). HNF4 is not present in the latter database. Only transcription factors with a minimum of 10 target genes in signature were analysed. (DOC) [file pone.0023365.s004.doc]

**Table S4.** Transcription factor predictions by TFactS

| Transcription factor | E.value  (corrected p-value) | Number of target genes in intersection | Total number of target genes in signature | Regulation  (if significant) | Number of genes matching the regulation |
| --- | --- | --- | --- | --- | --- |
| LXR | <10^-5^ | 7 | 19 | Activated | 7 |
| SREBP | <10^-5^ | 19 | 46 | Activated | 19 |
| PPARα | <10^-5^ | 10 | 20 | Inhibited | 5 |
| FOXO1 | 2,2.10^-3^ | 8 | 145 | Inhibited | 7 |
| HNF4A | 4,0.10^-2^ | 5 | 78 | - | - |

The list of genes changed in n-3 PUFA depleted mice compared to control mice (in the fed and starved conditions) was submitted to TFactS (sign-less) using default settings. Transcription factors that are significantly regulated with p-value, E-value, Q-value, FDR and random control <5% are shown. The regulation type (activation or inhibition) is indicated if it was found significant (p<0.05) by TFactS (sign-sensitive). HNF4 is not present in the latter database. Only transcription factors with a minimum of 10 target genes in signature were analysed.
