# Supplementary material for: Hepatic n-3 Polyunsaturated Fatty Acid Depletion Promotes Steatosis and Insulin Resistance in Mice: Genomic Analysis of Cellular Targets
Source: PLoS One. 2011 Aug 10;6(8):e23365. doi: 10.1371/journal.pone.0023365 (PMC3154437; doi:10.1371/journal.pone.0023365)
Supplement: Table S5 — qPCR confirmation of genes modulated in the liver of n-3 PUFA depleted mice (DEF) compared to control mice (CT). mRNA content of sterol-regulatory-element-binding protein-1c (SREBP-1c), fatty acid synthase (FAS), stearoyl-CoA desaturase-1 (SCD-1), ATP-binding cassette transporters G5 (ABCG5), insulin induced gene 2a (Insig2a), carbohydrate-responsive element-binding protein (Chrebp), liver-pyruvate kinase (L-PK), cytochrome P450, family 7, subfamily A, polypeptide 1, (cyp7a1) and 3-hydroxy-3-methyl-glutaryl-CoA reductase (HMGCoAr) in the liver of mice fed a control (CT; n = 6) or n-3 PUFA depleted diet (DEF; n = 7) for three months. Results were analysed according to the 2−ΔΔ ct method and were normalised to RPL-19 mRNA. Data are the mean ± SEM. *: mean values significantly different (P<0.05, Student t-test) (DOC) [file pone.0023365.s005.doc]

**Table S5.** qPCR confirmation of genes modulated in the liver of n-3 PUFA depleted mice (DEF) compared to control mice (CT)

| Relative expression | CT | DEF |
| --- | --- | --- |
| SREBP-1c | 1.03 ± 0.11 | 1.85 ± 0.36 |
| FAS | 1.02 ± 0.09 | 2.46 ± 0.22* |
| SCD-1 | 1.16 ± 0.27 | 2.44 ± 0.46* |
| ABCG5 | 1.02 ± 0.08 | 1.28 ± 0.06* |
| Insig2a | 1.11 ± 0.23 | 2.12 ± 0.46 |
| Chrebp | 1.02 ± 0.08 | 1.10 ± 0.07 |
| L-PK | 1.05 ± 0.14 | 2.10 ± 0.28* |
| Cyp7a1 | 1.10 ± 0.21 | 1.67 ± 0.26 |
| HMGCoAr | 1.05 ± 0.13 | 1.46 ± 0.11 |

mRNA content of sterol-regulatory-element-binding protein-1c (SREBP-1c), fatty acid synthase (FAS), stearoyl-CoA desaturase-1 (SCD-1), ATP-binding cassette transporters G5 (ABCG5), insulin induced gene 2a (Insig2a), carbohydrate-responsive element-binding protein (Chrebp), liver-pyruvate kinase (L-PK), cytochrome P450, family 7, subfamily A, polypeptide 1, (cyp7a1) and 3-hydroxy-3-methyl-glutaryl-CoA reductase (HMGCoAr) in the liver of mice fed a control (CT; n = 6) or n-3 PUFA depleted diet (DEF; n = 7) for three months.

Results were analysed according to the 2^-∆∆ ct^ method and were normalised to RPL-19 mRNA. Data are the mean ± SEM.

^*^: mean values significantly different (P<0.05, Student *t*-test)
