# Supplementary material for: Hepatic n-3 Polyunsaturated Fatty Acid Depletion Promotes Steatosis and Insulin Resistance in Mice: Genomic Analysis of Cellular Targets
Source: PLoS One. 2011 Aug 10;6(8):e23365. doi: 10.1371/journal.pone.0023365 (PMC3154437; doi:10.1371/journal.pone.0023365)
Supplement: Table S6 — Composition of control (CT) and n-3 PUFA depleted (DEF) diet. Formulated by Research Diets. Parenthetical numbers indicate the manufacturer's diet number. (DOC) [file pone.0023365.s006.doc]

**Table S6.** Composition of control (CT) and n-3 PUFA depleted (DEF) diet

| Composition | CT (D08041805) | DEF (D08041806) |
| --- | --- | --- |
| Casein (%) | 20 | 20 |
| Corn starch (%) | 44.2 | 44.2 |
| Sucrose (%) | 10 | 10 |
| Maltodextrin (%) | 13.2 | 13.2 |
| Cellulose (%) | 5 | 5 |
| Soybean oil (%) | 5 | 0 |
| Sunflower oil (%) | 0 | 5 |
| Mineral mix (%) | 3.5 | 3.5 |
| Vitamin mix (%) | 1 | 1 |
| n-3/n-6 PUFA | 0.145 | 0.008 |

Formulated by Research Diets. Parenthetical numbers indicate the manufacturer's diet number.
