# Supplementary material for: Hepatic n-3 Polyunsaturated Fatty Acid Depletion Promotes Steatosis and Insulin Resistance in Mice: Genomic Analysis of Cellular Targets
Source: PLoS One. 2011 Aug 10;6(8):e23365. doi: 10.1371/journal.pone.0023365 (PMC3154437; doi:10.1371/journal.pone.0023365)
Supplement: Table S7 — Fatty acid composition of soybean and sunflower oil. Fatty acid composition of soybean and sunflower oil used for the CT and DEF diet respectively. (DOC) [file pone.0023365.s007.doc]

**Table S7.** Fatty acid composition of soybean and sunflower oil

| Composition g/100g of oil | soybean | sunflower |
| --- | --- | --- |
| C16:0 | 10.4 | 6.6 |
| C18:0 | 3.8 | 5.1 |
| C20:0 | 0 | 0.5 |
| C16:1 | 0 | 0.5 |
| C18:1 | 24.4 | 23.1 |
| C18:2 n-6 | 53.6 | 63.7 |
| C18:3 n-3 | 7.8 | 0.5 |

Fatty acid composition of soybean and sunflower oil used for the CT and DEF diet respectively.
