# Supplementary material for: Hepatic n-3 Polyunsaturated Fatty Acid Depletion Promotes Steatosis and Insulin Resistance in Mice: Genomic Analysis of Cellular Targets
Source: PLoS One. 2011 Aug 10;6(8):e23365. doi: 10.1371/journal.pone.0023365 (PMC3154437; doi:10.1371/journal.pone.0023365)
Supplement: Table S8 — Primers sequences used for real-time quantitative PCR. RPL19; ribosomal protein L19, FAS; fatty acid synthase, SREBP-1c; sterol-regulatory-element-binding protein-1c, Chrebp; carbohydrate-responsive element-binding protein, L-PK; liver-pyruvate kinase, SCD-1; stearoyl-CoA desaturase-1, ATP-binding cassette transporters G5; ABCG5, cyp7a1; cytochrome P450, family 7, subfamily A, polypeptide 1, Insig2a; insulin induced gene 2a, HMGCoAr; 3-hydroxy-3-methyl-glutaryl-CoA reductase, EDEM1; ER degradation enhancer, mannosidase alpha-like 1, spliced (s) and unspliced (u) XBP-1; X-box binding protein 1, GRP78; glucose-regulated protein 78, GRP94; glucose-regulated protein 94, CHOP; CCAAT/enhancer binding protein homologous protein, PDI; protein disulfide isomerise. (DOC) [file pone.0023365.s008.doc]

**Table S8.** Primers sequences used for real-time quantitative PCR

|  | Forward primer (5’ to 3’) | Reverse primer (5’ to 3’) |
| --- | --- | --- |
| RPL-19 | GAAGGTCAAAGGGAATGTGTTCA | CCTTGTCTGCCTTCAGCTTGT |
| FAS | TTCCAAGACGAAAATGATGC | AATTGTGGGATCAGGAGAGC |
| SREBP-1c | GATCAAAGAGGAGCCAGTGC | TAGATGGTGGCTGCTGAGTG |
| Chrebp | CTGGGGACCTAAACAGGAGC | GAAGCCACCCTATAGCTCCC |
| L-PK | CTTGCTCTACCGTGAGCCTC | ACCACAATCACCAGATCACC |
| SCD-1 | CCTCTTCGGGATTTTCTACTACATG | GCCGTGCCTTGTAAGTTCTGT |
| ABCG5 | TGGCCCTGCTCAGCATCT | ATTTTTAAAGGAATGGGCATCTCTT |
| Cyp7a1 | GGGATTGCTGTGGTAGTGAGC | GGTATGGAATCAACCCGTTGTC |
| Insig2a | TGTGAGCTGGACTAGCTTGCT | CCTAAGCCGTAAAACAAAATG |
| HMGCoAr | CCTGACACTGAACTGAAGCG | TCTTTCCAGAACACAGCACG |
| EDEM1 | CAGACGAGCTGTGAAAGCCC | AACCCAATGGCCTGTCTGG |
| sXBP-1 | GAGTCCGCAGCAGGTG | GTGTCAGAGTCCATGGGA |
| uXBP-1 | AAGAACACGCTTGGGAATGG | ACTCCCCTTGGCCTCCAC |
| GRP78 | TGCAGCAGGACATCAAGTTC | GTTTGCCCACCTCCAATATC |
| GRP94 | AAACGGCAACACTTCGGTCAG | GCATCCATCTCTTCTCCCTCATC |
| CHOP | CCTAGCTTGGCTGACAGAGG | CTGCTCCTTCTCCTTCATGC |
| PDI | ACAGCTGGCAGGGAAGCTGA | AGCCTCTGCTGCCAGCAAGA |

RPL19; ribosomal protein L19, FAS ; fatty acid synthase, SREBP-1c ; sterol-regulatory-element-binding protein-1c, Chrebp ; carbohydrate-responsive element-binding protein, L-PK ; liver-pyruvate kinase, SCD-1; stearoyl-CoA desaturase-1, ATP-binding cassette transporters G5; ABCG5, cyp7a1 ; cytochrome P450, family 7, subfamily A, polypeptide 1, Insig2a; insulin induced gene 2a, HMGCoAr; 3-hydroxy-3-methyl-glutaryl-CoA reductase, EDEM1; ER degradation enhancer, mannosidase alpha-like 1, spliced (s) and unspliced (u) XBP-1; X-box binding protein 1, GRP78; glucose-regulated protein 78, GRP94; glucose-regulated protein 94, CHOP ; CCAAT/enhancer binding protein homologous protein, PDI; protein disulfide isomerise.
